# Growth and micronutrient status parameters of Nigerian preterm infants consuming preterm formula or breastmilk

**DOI:** 10.1038/s41390-023-02976-6

**Published:** 2024-01-09

**Authors:** Adedotun Joshua Owolabi, Idowu Adejumoke Ayede, Olugbenga Oyewumi Akinrinoye, Adegoke Gbadegesin Falade, Gboyega Bosun Ajibola, Ologunore Olufisayo Christopher, Gregory Olawole Arifalo, Ayodele Oladejo Abiona, Edith J. M. Feskens, Alida Melse-Boonstra, Anne Schaafsma

**Affiliations:** 1 Division of Human Nutrition and Health, Wageningen University and Research, P.O. Box 9101, 6700 HB Wageningen, The Netherlands; 2 Department of Pediatrics, University College Hospital, Ibadan, Nigeria; 3 Department of Obstetrics and Gynaecology, University College Hospital, Ibadan, Nigeria; 4 Department of Family Medicine, Sacred Heart Hospital, Lantoro, Abeokuta, Nigeria; 5 Department of Pediatrics, Adeoyo Maternity Teaching Hospital, Ibadan, Nigeria; 6 Expert Nutrition Team, FrieslandCampina, Stationsplein, Amersfoort, The Netherlands

## Abstract

**Background:**

Moderate-to-late preterm infants (32–34 weeks GA) have increased risk of neonatal morbidities compared to term infants, however dedicated nutritional guidelines are lacking.

**Methods:**

Moderate-to-late preterm infants received a preterm formula (*n* = 17) or breastmilk (*n* = 24) from age 2–10 weeks in a non-randomized, open-label observational study. Anthropometric measurements were assessed bi-weekly. Blood concentrations of hemoglobin, ferritin, serum retinol, and 25-hydroxy-vitamin D (25OHD) were analyzed at age 2 and 10 weeks.

**Result:**

Average growth per day was 14.7 g/kg BW/day in formula-fed and 12.8 g/kg BW/day in breastmilk-fed infants but not different from each other. Length and head circumference in both groups were in line with the median reference values of the Fenton growth chart. At 10 weeks of age, hemoglobin tended to be higher in the formula-fed group (10.2 g/dL vs. 9.6 g/dL, *p* = 0.053). 25OHD increased in formula- and breastmilk-fed infants from 73.8 to 180.9 nmol/L and from 70.7 to 97.6 nmol/L, respectively. Serum retinol only increased in the formula-fed group (0.63 to 1.02 µmol/L, *p* < 0.001).

**Conclusion:**

Breastfeeding resulted in adequate growth in moderate-late preterm infants but was limiting in some micronutrients. The preterm formula provided adequate micronutrients, but weight gain velocity was higher than the Fenton reference value.

**Impact statement:**

- Unfortified breastmilk resulted in adequate growth in weight, length and head circumference in Nigerian moderate to late preterm infants during an study period of 8 weeks, but status of vitamin D, vitamin A and iron needs to be monitored.
- The high-energy formula, developed for very preterm infants, resulted in higher growth in body weight in moderate to late preterm infants than the median of the Fenton preterm growth chart.
- This study supports the necessity of dedicated nutritional guidelines, and regular monitoring of growth and nutritional status of moderate to late preterm infants.

## Introduction

Annually, 800,000 preterm infants are born in Nigeria, of whom 85% are moderately (32–33 weeks gestational age [GA]) or late preterm (34–36 weeks GA). Although being considered relatively healthy, these moderate-to-late preterm infants still have an increased risk of neonatal morbidities and retarded development as compared to term-born infants.^1–7^ However, only for the extremely and very preterm infants dedicated nutritional guidelines have been developed.^8,9^

The main target for feeding of preterm infants is to maintain, or catch up with, normal fetal growth rates without metabolic stress, and to prevent micronutrient deficiencies. For monitoring growth, the Fenton preterm growth chart is the most widely used, allowing growth monitoring of preterm infants from as early as 22 weeks GA to 10 weeks of post-term age.^10,11^ Recently a survey among healthcare professionals from seven countries showed that a majority (~60%) of respondents rated unfortified human milk as adequate for growth of late preterm infants, and >70% of them considered good growth as a weight gain of 15–20 g/kg/day in-hospital and >20 g/kg/day after discharge.^12^ However, according to Fenton preterm growth chart, normal weight gain velocity for late preterm infants at the age of 34 weeks is 15 g/kg/day, decreasing to approximately 8 g/kg/day at term age, and 4–5 g/kg/day at 50 weeks.^10,13^

The European Society of Pediatric Gastroenterology Hepatology and Nutrition (ESPGHAN) also considers unfortified breastmilk as sufficient for appropriate for gestational age (AGA) late preterm infants. The increased nutritional needs of these infants can be compensated by an increased intake of breastmilk.^14^ With regard to formula feeding, preterm infant formulas used in hospitals are based on the recommendations as set for extremely and very preterm infants.^8^ Use of these typical preterm infant formulas for a longer period may lead to increased weight gain in the form of fat mass which is associated with health risks at later age.^15^

Regarding nutrient requirements, adequate intake of protein is particularly important for preterm infants accelerated growth. The recommended amount of protein intake for infants with a GA of 32–37 weeks is 2.5–3.5 g/kg/day, decreasing to 2–3 g/kg/day at term.^15–17^ The protein-energy ratio (2.5–3 g/100 kcal) is considered to be important to realize qualitative growth instead of stimulating fat mass deposition,^15,17,18^ although this ratio is not achieved in human milk. Concerning micronutrients, iron, vitamin D, and vitamin A are often limited in preterm born infants.^8,14^ The risk of developing iron deficiency is mainly caused by limited iron stores at birth and fast postnatal growth.^14^ Vitamin D status at birth is positively related to the length of gestation and maternal vitamin D status.^19,20^ For both iron and vitamin D the concentrations in breastmilk are low.^21,22^ Of the reported normal vitamin A range (18–60 µg-RE/100 ml) in mature human milk (term as well as preterm) only the higher concentrations can be considered as sufficient for term infants (390 µg-RE per day).^8,23^

In the present study, growth and the nutritional status of iron, and vitamins A and D were studied in moderate-to-late Nigerian preterm infants fed with a preterm infant formula or unfortified breastmilk from 2 to 10 weeks of age.  The objective of this study is premised on the assumption that moderately late preterm infants fed with higher calorie density will grow faster than breastfed moderately late preterm infants and that preterm formula will supply sufficient vitamins and minerals. 

## Methods

### Study design

In this non-randomized observational study, healthy moderate-to-late preterm infants (32–34 weeks gestational age) were recruited from three hospitals in Nigeria: the University College Hospital (Ibadan, Oyo State), Adeoyo Maternity Teaching Hospital (Ibadan, Oyo State), and Sacred Heart Hospital (Lantoro, Abeokuta, Ogun State). Over the initial 14 postnatal days, parents were informed about the study by medical research teams, and informed consent was obtained for infant participation. Eligible infants were then enrolled and provided with either their mother’s milk or, when medically necessary, a preterm formula (Peak Baby Preterm, FrieslandCampina, Nigeria). The formula’s nutritional composition is detailed in Supplementary Table S1. Medical indications for formula use were determined by local pediatricians or neonatologists following standard care protocols, including insufficient breastmilk due to multiple births or orphan status.

The study period spanned 8 weeks (from ages 2 to 10 weeks) or until infants reached a weight of 3500 g. The study received approval from the University of Ibadan College Hospital ethics committee (assigned number UI/EC/16/0418) and was registered in the Netherlands trials register under NTR6156 at https://trialsearch.who.int/.

### Subjects

Eligible healthy moderate-to-late preterm infants (GA 32–34 weeks) had to be 2–3 weeks of age at inclusion, AGA, and on full enteral feeding. In the formula group, at least 50% of daily milk intake had to be as preterm formula at inclusion and 100% at 4 weeks of age. In the case of breastmilk, at least 75% of daily milk intake had to be breastmilk. Infants with congenital malformations, conditions known to affect growth (e.g., severe bronchopulmonary dysplasia, inborn errors of metabolism, cardiac or renal disease, necrotizing enterocolitis with substantial gut loss, and grade IV intraventricular hemorrhage), family history of impaired iron metabolism (haptoglobin Hp2-2, hemochromatosis, sickle cell anemia, thalassemia), or medications that could affect digestion and or absorption of food or sleep were not included in the study. Furthermore, blood transfusions and vitamin supplements were not allowed during the study period.

Body weight, recumbent length, and head circumference (HC) were measured by trained pediatricians and research assistants using calibrated equipment. Growth data were collected at the start of the study (age 14 ± 2 days), and every 14 days thereafter until age 75 ± 2 days. Most of these measurements took place at the homes of participants. The equipment for body weight (Seca 834 Electronic baby and child scales) and recumbent length (Seca 417 mobile Infantometer) were calibrated every day by using a known weight of 2 kg or a 40 cm length standard. Infants were weighed wearing only a dry diaper, whereas length was measured naked, and knees were gently pressed towards the board in order to fully extend the feet. Each measurement was done three times and the mean value was recorded. The difference between two measurements had to be <10 g or <0.5 cm. For weight gain velocity, the Average 2-point method (Avg2pt) was used with initial weight at baseline (W1) and weight at endline (W2) as a function of time: *((W2* *–* *W1)/([(W2* + *W1)/2] /1000))/ number of days.*^13^ Growth in gram per day was calculated by: (weight endline-weight start)/56 days. Z-scores and small for gestational age (SGA) or AGA classifications were calculated using the Fenton z-score calculator (http://ucalgary.ca/fenton).^10^

Head circumference (HC) measurements were taken using a flexible, non-stretchable tape (Seca 212 Measuring tape). The tape was positioned around the largest part of the head, with the lower edge just above the eyebrows and ears, encircling the largest part of the back of the head. Measurements were recorded to the nearest 0.1 cm, with each infant being measured three times, and the mean value was recorded. All outcomes were compared to median growth values obtained from the Fenton preterm growth chart or data in this manuscript.^13^

Venous blood samples (5 ml in total) were collected at the study’s outset to assess Hb, serum ferritin, 25OHD, and serum retinol. These frozen serum and plasma samples were shipped to the Netherlands on dry ice with temperature monitoring and analyzed at the Amsterdam University Medical Centre. Vitamin D (plasma 25OHD2 and 25OHD3) was determined in EDTA plasma using an optimized LC-MS/MS method described by Dirks et al (referring to method E).^24^ For this study, two cut-off values for 25OHD are considered: 50 (skeletal metabolism) and 75 nmol/L (extra-skeletal activities).^25–27^ Heparin plasma was used for the analysis of retinol (vitamin A). Retinol was determined using isocratic high-performance liquid chromatography with UV detection,^28^ with a sufficiency cut-off value of 0.7 µmol/L (200 µg/L).^29^ Serum ferritin was analyzed using Particle Enhanced Immunoturbidimetric Assay (Cobas c 502 analyzer, Roche/Hitachi, Mannheim, Germany), with a cut-off value of 76 µg/L.^30^ Hb was measured in whole blood according to standard procedures at the University of Ibadan Clinical Laboratory (Ibadan, Nigeria) with a cut-off value of 10 g/dL.^31^

The daily amount of formula milk consumed was recorded by the nurse or mother. Furthermore, the number of hospital days was monitored.

Breast milk samples (30 ml in total) were taken at lactation day 45 ± 2, between 12–14 h o’clock, from a full expression of one breast. Aliquots were frozen at −20 °C and transported to the Netherlands on dry ice, and analyzed for fatty acids and vitamin D (University Medical Center Groningen, Groningen, The Netherlands), iron, and vitamin A, using methodology described earlier.^21,32^ Vitamin D is expressed as international units of anti-rachitic activity (ARA) in which 25 ng/L parent vitamin D2-D3, or 5 ng/L 25OHD are calculated as 1 IU ARA.^33^ Retinol and iron concentrations were analyzed by the European Laboratory of Nutrients (Bunnik, The Netherlands), using ICP-MS for iron and LC-MS-MS for retinol.

Finally, tolerance parameters (reflux, cramps/colic, stool consistency/frequency/color) were recorded at the start of the study, and at 45 ± 2 and 75 ± 2 days of age, using a 3-day recall questionnaire (filled in just before the scheduled home or hospital visits) (Supplementary Fig. s1).

### Statistics

The sample size estimation was based on findings from previous research,^34^ in which a comparable group of Dutch preterm formula-fed infants showed a higher increase in daily body weight (on average 4.5 g/kg BW/d, *p* < 0.015) when the protein content of the formula was higher (1.6 vs. 1.9 g/100 ml, 75 kcal). In breastmilk, the protein content is about 1 g/100 ml (~65 kcal), whereas the present study, formula provided 2.56 g protein per 100 ml (80 kcal), hence the differences in energy and protein intake in the current study were considerably larger and 30 infants per group, as in the previous study, was considered sufficient (with an estimated power >90%, using ClinCalc.com Sample Size Calculator) to find a difference in weight gain’.

Growth data, absolute values and delta values with 5 and 4 repeated measurements respectively, were analyzed using three-way mixed ANOVA (BBW) with time as within-group variable, and sex and group as between-group variables. Interactions were studied for time*group*sex, time*group, time*sex, and group*sex. GLM univariate analysis of variance was used to study the effects per time point for each significant interaction. For growth velocities, generalized estimated equations (GEE) was used since these data were often nonparametric.

All other normally distributed data were tested with paired or unpaired T-tests to study differences within or between groups. When co-variables had to be considered, one-way ANCOVA was used. In case of skewed data, comparable non-parametric tests were used.

The statistical evaluation took place by using IBM SPSS 26 (IBM, IBM, Armonk, NY). A *p*-value of ≤0.05 was statistically significant, a *p*-value of <0.1 was considered to show a trend.

## Results

### Participants

**Fig. 1:**
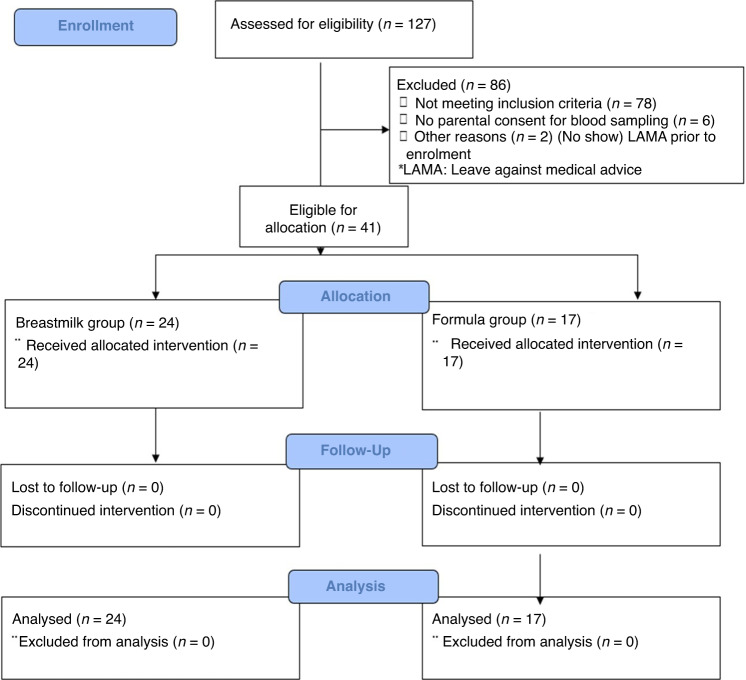
Flowchart of screening and allocation. Flowchart of screening and allocation to study groups of moderate–late preterm Nigerian infants.

The main reasons for recruiting fewer infants than expected (Fig. 1) were three long hospital strikes that restricted neonatal admissions, refusal of parents because of blood sampling, several SGA infants at birth, and an extension to other hospitals that came too late. A strict breastfeeding policy and providing formula only on medical prescription did limit the inclusion of formula-fed infants. Reasons for the formula prescription were maternal death (*n* = 1), no or insufficient breast milk available (*n* = 16) especially in case of multiple births (*n* = 12; Table 1). Seven SGA infants (2 on breastmilk, 5 on formula) were included based on the advice of the principal investigator. All infants finalized the study.

**Table 1:** General characteristics of study participants.

Parameter	Formula-fed infants (*N* = 17)	Breastmilk-fed infants (*N* = 24)	*P*-value
Boys/girls	8/9	8/16	0.518^*^
Gestational age	33.3 ± 0.8	32.6 ± 0.7	0.026
Birthweight (g)			
All	1650.0 ± 233.9	1702.0 ± 167.1	0.438^**^
Boys	1600.0 ± 223.6	1768.7 ± 166.8	0.747^**^
Girls	1694.4 ± 246.8	1668.7 ± 162.1	0.098^**^
SGA^a^			
birth	5 (2 girls, 3 boys)	2 (2 girls)	0.077
Age at inclusion (weeks)	1.96 ± 0.8	2.0 ± 0.20	0.142^**^
Singletons	5	20	0.001^*^
Twins	1	2	
Triplets	2		
Quadruplets	1		
Number of hospital days: mean ± SD (median; range)			
All	12.4 ± 2.8 (13; 5–17)	13.4 ± 7.8 (12; 5–47)	0.523^**^
Boys	13.1 ± 2.0 (13; 11–17	12.0 ± 3.6 (12; 5–18)	0.456^**^
Girls	11.8 ± 3.5 (14; 5–14)	14.2 ± 9.3 (12; 8–47)	0.344^**^

Data are provided as mean ± SD, or absolute numbers. Z-score calculator based on the Fenton preterm growth chart.^10^

*SGA* small for gestational age.

^*^Fisher’s exact test, 2-sided; ^**^Unpaired T-test.

### Product intake

Median milk intake in the formula group was 115 ml/kg BW/day at age 2 weeks, increasing to 152 ml at age 4 weeks, and to 165 ml at 8 weeks of age. At 10 weeks of age, the intake dropped again to 147 ml. Average consumption of formula tended to be higher in girls than in boys at 4 (*p* = 0.060) and 10 weeks (*p* = 0.056) of age. Girls in the formula group consumed 15–35% more volume of formula than instructed. None of the formula-fed infants received breastmilk during the study period. Breastmilk-fed infants did not receive any formula. Although vitamin supplements were not allowed during the study period, most infants did receive one or a combination of supplements that included iron, vitamin A and/or vitamin D2 (Supplementary Table s2).

### Anthropometry

#### Body weight

**Fig. 2:**
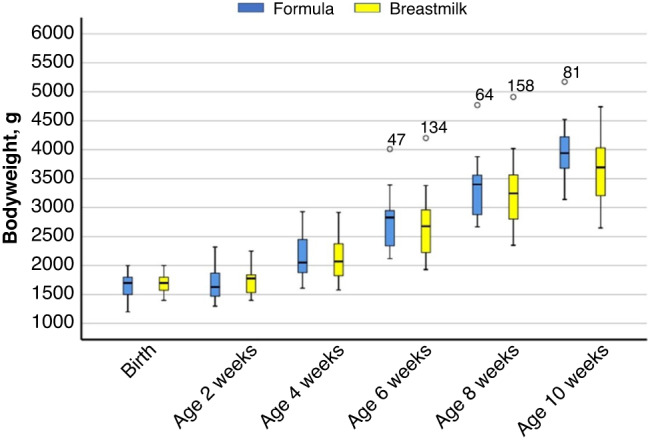
Body weight development (g) of Nigerian moderate–late preterm born infants on full enteral feeding with breast milk (*n*=24) or special preterm formula (*n*=17), from 2 to 10 weeks of age. Data are presented as boxplots (median, first quartile at the lower boundary, third quartile at upper boundary, and whiskers representing the minimum and maximum values within a distance of 1.5 inter-quartile range value. Values outside this range are reported separately).

Within each group, a significant linear improvement of weight was shown (*p* < 0.001) during the study period (Fig. 2). In the breast milk group, sex affected body weight in week 10 (*p* = 0.046) and tended to do so in week 6 (*p* = 0.057): boys weighed 446 ± 216 (SE) g less than girls in week 10, and 400 ± 203 g less in week 6. The three-way interaction between time, group and sex (data not shown) was statistically significant (*p* = 0.029), whereas a significant two-way interaction was found for sex and group (*p* = 0.017). A trend towards significance was found for time*group (*p* = 0.053). Table 2 shows body weights at base and endline of the study as well as the average daily growth in grams.

**Table 2:** Body weights, and changes in body weights (total weight gain, g/day), in Nigerian late preterm infants who were either breastmilk or preterm formula-fed.

Parameter	Formula *N* = 17	Breastmilk *N* = 24	*P* value (between groups)^b^
Body weight at 2 weeks of age (g)			
All	1668.0 ± 283.8	1730.0 ± 227.3	*0.463*
Boys^a^	1561.2 ± 247.8	1833.8 ± 242.0	***0.043***
Girls^a^	1763.3 ± 292.8	1678.1 ± 208.0	*0.455*
*Within the group*^b^*, p* =	*0.148*	*0.116*	
Body weight at 10 weeks of age (g)			
All	3972.9 ± 479.1	3681.3 ± 552.4	*0.080*
Boys^a^	3802.5 ± 432.7	3978.8 ± 488.5	*0.458*
Girls^a^	4124.4 ± 490.3	3532.5 ± 534.5	***0.012***
*Within the group*^b^*, p* =	*0.174*	*0.060*	
Weight gain (g) from 2 to 10 weeks of age			
All	2307.7 ± 305.2	1951.3 ± 431.9	***0.004***
Boys^a^	2241.3 ± 266.4	2145.0 ± 382.6	*0.570*
Girls^a^	2361.1 ± 341.4	1854.0 ± 433.2	***0.004***
*Within the group*^b^*, p* =	*0.437*	*0.122*	
% Weight gain (g) from 2 to 10 weeks of age	*138%*	*113%*	
Growth in g/day			
All	41.2 ± 5.5	34.8 ± 7.7	***0.004***
Boys^a^	40.0 ± 4.8	38.3 ± 6.8	*0.570*
Girls^a^	42.2 ± 6.1	33.1 ± 7.7	***0.004***
*Within the group*^b^*, p* =	*0.437*	*0.122*	

^a^Data are mean ± SD. In the formula group 8 boys and 9 girls participated, whereas for the breastmilk these figures are 8 and 17, respectively.

^b^Independent Samples T-test.

Total weight gain during the study period, and the average growth per day appeared to be different between groups (3973 g formula-fed vs. 3681 g breastfed, *p* = 0.080), and this was mainly caused by the growth of girls. However, when correcting for multiple births these statistical differences disappeared.

Average weight gain velocity in both groups was at all times well above the median values reported for the specific postconceptional ages.^13^ For the average weight gain velocity during the total study period, 94.1% of formula-fed infants (average growth 14.7 ± 1.53 g/kg BW/day) and 87.5% of breastmilk-fed infants (average growth 12.8 ± 1.77 g/kg BW/day) were above the expected median weight gain velocity of 10.5 g/kg BW/day for 35–42 weeks of postconceptional age. Weight gain velocities between groups were not different (*p* = 0.168) when corrected for multiple births. Girls in the formula group grew faster than their breastmilk-fed counterparts, whereas formula-fed boys showed an almost equal growth as compared to boys in the breastmilk group.

**Fig. 3:**
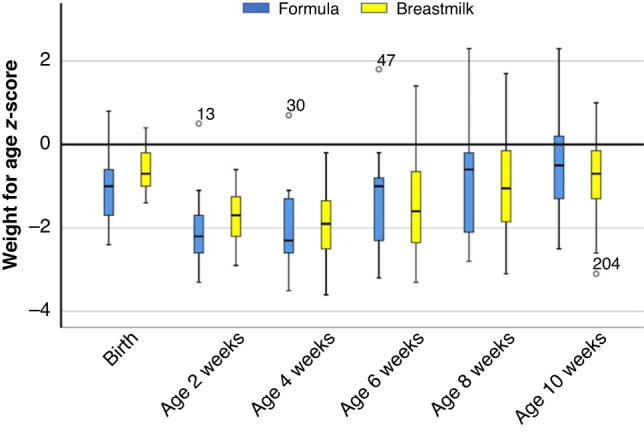
Weight-for-age z-scores among late preterm-born infants fed with breast milk (*n*=24) or preterm formula (*N*=17), from birth −to 10 weeks of age. Data are presented as boxplots (median, first quartile at the lower boundary, third quartile at upper boundary, and whiskers representing the minimum and maximum values within a distance of 1.5 inter-quartile range value. Values outside this range are reported separately). Z-scores were calculated using the *z*-score calculator developed by Fenton TR.^11^ The groups did not show statistically significant differences.

Both groups showed an increase in weight-for-age z-score (WAZ) during the study period. Breastmilk-fed infants with a WAZ at birth of −0.63 ± 0.50, decreased to WAZ −1.71 ± 0.58 at 2 weeks of age but ended up with a WAZ of −0.81 ± 1.06 at 10 weeks of age. For the formula-fed infants, these WAZ were −1.02 ± 0.80, −2.08 ± 0.93, and −0.49 ± 1.12, respectively (Fig. 3).

#### Body length

Both group showed a significant linear improvement (*p* < 0.001) in length (formula-fed infants from 42.94 ± 2.08 to 52.47 ± 2.24 cm, and breastmilk-fed infants from 42.58 ± 2.39 to 52.42 ± 1.60) without differences between the groups. At the ages of 2 and 10 weeks (Table 3), boys in the formula-fed group were shorter than their breast-fed counterparts (*p* < 0.042), whereas girls in the formula-fed group were taller than breastfed girls (*p* < 0.017). The average increase in length per week (formula-fed: 1.19 ± 0.08, breastmilk-fed: 1.23 ± 0.20 cm/week) was not different between the groups (for all infants as well as for each of the sex), and slightly higher than the expected median growth value (1.03 cm/week) for 35–42 weeks postconceptional age.^13^ However, there tend to be a borderline interaction between time*sex (*p* = 0.055) and time*sex*group product (*p* = 0.056).

**Table 3:** Length at the age of 2 and 10 weeks in Nigerian late preterm infants fed either breast milk on special preterm formula.

Parameter	Formula-fed *N* = 17	Breastmilk-fed *N* = 24	*P value*^a^
Length at 2 weeks of age			
All	42.94 ± 2.08	42.58 ± 2.39	*0.613*
Boys	41.88 ± 2.10	44.38 ± 2.07	***0.031***
Girls	43.89 ± 1.62	41.69 ± 2.06	***0.008***
Length at 10 weeks of age			
All	52.47 ± 2.24	52.42 ± 1.60	*0.943*
Boys	51.38 ± 2.39	54.25 ± 2.71	***0.041***
Girls	53.44 ± 1.67	51.50 ± 1.90	***0.016***
% length growth from 2 to 10 weeks of age	22%	23%	

Data are presented as mean ± SD.

^a^Independent Samples T-test.

#### Head circumference

Both groups of infants showed a significant linear growth in HC (*p* < 0.001) for the total study period (formula-fed from 30.88 ± 1.50 to 37.18 ± 1.29 cm, and for breastmilk-fed from 30.63 ± 1.35 to 36.88 ± 1.42 cm). At 2 and 10 weeks, the HC of girls in the formula-fed group was larger as compared to girls in the breastfed group (*p* = 0.037). The average increases in HC (cm/week) during the study were not different between groups (formula-fed: 0.79 ± 0.09, breastmilk-fed: 0.78 ± 0.11 cm/week) and higher than the expected median growth value (0.58 cm/week) for 35–42 weeks postconceptional age.^13^ Changes in head circumference for both groups are reported in Table 4.

**Table 4:** Changes in head circumference for the breastfed milk (*N* = 24) and formula-fed (*N* = 17) moderate-late preterm Nigerian infants, as well as per sex total and according to sex, from the start of the study (age 2 weeks) till the age of 10 weeks of age.

Parameter	Formula-fed *N* = 17	Breastmilk-fed *N* = 24	*P* value^a^
Head circumference at age 2 weeks			
All	30.88 ± 1.50 30.00 (2.00)	30.63 ± 1.35 31.00 (1.00)	*0.634*
Boys	30.13 ± 1.13 30.00 (2.00)	31.25 ± 1.75 31.00 (2.75)	*0.161*
Girls	31.56 ± 1.51 32.00 (2.00)	30.31 ± 1.01 30.50 (1.00)	***0.037***
Head circumference at age 10 weeks			
All	37.18 ± 1.29 36.50 (2.50)	36.88 ± 1.42 37.00 (2.00)	*0.361*
Boys	36.50 ± 1.20 36.50 (2.50)	37.63 ± 1.77 37.50 (3.25)	*0.234*
Girls	37.78 ± 1.09 38.00 (1.00)	36.50 ± 1.10 37.00 (1.75)	***0.017***
% Head Circumference growth from 2 to 10 weeks of age	20%	20%	

Data were in part non-parametric and thus presented as mean ± SD as well as median (IQR).

The average increases in HC (cm/week) during the study were not different between groups (formula-fed: 0.79 ± 0.09, breastmilk-fed: 0.78 ± 0.11 cm/week) and higher than the median growth value (0.58 cm/week) as reported for 35–42 weeks postconceptional age.^13^

^a^Mann–Whitney U-test.

### Nutritional parameters in blood

In both feeding groups, concentrations of Hb and serum ferritin (as parameters of iron status), decreased during the study period of 8 weeks. At the age of 10 weeks, mean concentrations of ferritin had decreased from 427.1 ± 174.5 to 158.5 ± 121.1 µg/L in breastmilk-fed infants (*p* = 0.001), and from 452.3 ± 177.8 to 115.8 µg/L in formula-fed infants (*p* = 0.003). In the breastmilk group, 67% of the infants were still above the minimum value of the reference range (≥76 µg/L ferritin) at the end of the study. For the formula-fed infants this percentage was 75%. Hb decreased from 13.3 ± 1.3 to 9.6 ± 1.3 g/dL in the breastmilk group (*p* < 0.001), and from 13.4 ± 2.2 to 10.2 ± 0.75 g/dL in the formula group (*p* = 0.001). Hb concentrations in the formula-fed group tended (*p* = 0.053) to be slightly higher at the end of the study than in the breastfed group. At 10 weeks of age, 62% of breastfed infants and 94% of formula-fed infants were at or above the minimum value of the reference range (≥10 g/dL).

In the breastfed group, 25OHD improved from 70.7 ± 17.4 nmol/L at 2 weeks of age to 97.6 ± 39.9 nmol/L at 10 weeks of age, with 75% of the infants above the minimum reference value (≥75 nmol/L 25OHD) at 10 weeks of age. Serum retinol did not improve in the breastmilk group, with 79% of infants being below the reference value of 0.7 µmol/L serum retinol at 10 weeks of age. Formula-fed infants significantly improved their vitamin D (25OHD) and vitamin A (serum retinol) status with 100% and 95% of the infants being above minimum reference values at 10 weeks of age. For 25OHD, the concentration increased from 73.8 ± 12.6 nmol/L at 2 weeks of age to 180.9 ± 173.5 nmol/L at 10 weeks of age (*p* < 0.001), while serum retinol increased from 0.63 ± 0.29 µmol/L to 1.02 ± 0.25 µmol/L (*p* < 0.001).

### Breastmilk composition

The measured concentrations of vitamin D (calculated as ARA), iron, and vitamin A in breastmilk were lower than those in the special preterm formula: ARA 13.1 ± 6.0 vs. 268 IU/100 ml, iron 0.025 ± 0.046 vs. 1.3 mg/100 ml, and retinol 6.8 ± 2.1 vs. 288 µg/100 ml. Levels of docosahexaenoic acid (DHA) and arachidonic acid (AA) in breastmilk were 0.66 ± 0.40 g% and 0.40 ± 0.14 g% of total fatty acids, respectively. In the formula these levels were 0.48 g% for both DHA and AA.

### Markers of food tolerance

No differences were seen in tolerance indicators (% reflux, number of defecations, feces color and consistency, % colic or cramps, belly distention, and % belly noise) between the groups (Supplementary Fig. s1).

### Number of hospital days

Most infants (16/17 in the formula group and 20/24 in the breastmilk group) were discharged from the hospital within 14 days postnatal. All other infants were discharged from the hospital at 3 weeks of age, except for one infant in the breastmilk group (discharged at 6.5/7 weeks). Body weight of most infants (15/17 in the formula group and 22/24 in the breastmilk group) at 14 days of age or at discharge was <2000 g (range at 14 days of age in the formula group 1300–2320 g, in the breast milk group 1400–2250 g).

## Discussion

Unfortified breastmilk sufficiently supported adequate growth in weight (12.8 ± 1.8 g/kg BW/day), length (1.23 ± 0.20 cm/week) and HC (0.78 ± 0.11 cm/week) in moderately-to-late preterm Nigerian infants during the study period at age 2–10 weeks. Preterm formula resulted in an average weight gain velocity (14.7 ± 1.53 g/kg BW/day) that was considerably higher than the reference median weight gain of 10.5 g/kg BW/day for infants with a postconceptional age of 35–42 weeks. However, at 10 weeks of age, body weights between the groups were not different (after correcting for birthweight) nor were total weight gain (g/day), average growth per day and weight gain velocity (g/kg/day) different between groups after correcting for multiple births. Average growth in length was not different between the groups, and slightly higher than the reference median growth reference values (1.03 cm/week for length and 0.58 cm/week for HC). Vitamin D and vitamin A status improved in the formula-fed infants. Although Hb and ferritin serum concentrations decreased, the majority (>75%) of formula-fed infants still had ≥10 g/dL Hb and ≥76 µ/L serum ferritin. In breastmilk-fed infants, vitamin D status also improved, but to a lesser extent as compared to the formula-fed infants. Vitamin A status in breastmilk-fed infants did not change, whereas Hb and ferritin concentrations decreased (>62% had ≥10 g/dl Hb and ≥76 µ/L ferritin). The effect on vitamin and mineral status might in part be caused by the supplements provided to most infants despite this was not allowed according to the study protocol.

Growth in weight was higher than expected when using the Fenton growth chart for preterm infants as a reference. It should be realized that the post-term growth information used in the Fenton chart derives from growth of breastmilk-fed as well as formula-fed infants in previous studies and at that time formula-fed infants were likely (also) not fed in the ideal way.^10,11^ In other words, the Fenton growth chart does not reflect optimal growth of late preterm infants, at least not post-term, but is the best reference available. The outcome of our observation is in line with the literature on formula-fed moderate-to-late preterm infants and is considered to be in particular an increase in fat mass.^14,35,36^ A high contribution of non-protein calories (>90 kcal/kg BW/day) could be a cause.^17^ Breastmilk would reach this level of non-protein calories (>90 kcal/kg BW/day) at an intake of 150 ml/kg BW/day), whereas in the formula of the present study this is reached at an intake of 130 ml/kg BW/day. Of interest to know and maybe in contrast to preterm infants, formula fed term infants showed to have a lower fat-mass at the ages of 3–4 months and 6 months, but not at 12 months, than breastmilk-fed infants.^37^ Overfeeding is common, and caregivers should be aware of and adhere to the recommended intakes unless there is a medical indication to do differently. Long-term effects of an early excessive increase in fat mass are not known, but may lead to an increased risk for obesity.^38^

Earlier reported growth rates of preterm low birth weight (LBW) Nigerian babies were 26.8–34 g/day, 0.86–0.96 cm/week for length, and 0.48–0.50 cm/week for HC in early infancy.^39^ Although the exact ages of these LBW infants are not reported, the average outcomes in the present study are within the upper range of these published figures, or higher (for formula and breastmilk fed infants respectively: weight 41.2 ± 5.4 and 35.0 ± 7.7 g/day; length 1.2 ± 0.1 and 1.2 ± 0.2 cm/week; HC 0.8 ± 0.1 and 0.8 ± 0.1 cm/week). A study in LBW infants from Chile, the UK and USA, showed that those fed a preterm formula weighed approximately 500 g more at term age than infants fed predominantly human milk. This absolute difference persisted until 6 months of corrected gestational age. Preterm formula infants were also longer and had larger HC at term than human milk-fed infants.^40^ Finally, it has been recommended that preterm infants should follow their own individual growth trajectory indicated by the birth weight percentile.^41^ When WAZ at birth should be regained postnatally, this was the case for the breastmilk group in the present study, but the formula-fed group ended up at a higher WAZ indicating growth beyond their ideal growth profile.

The formula used in the present study is in accordance with the ESPGHAN guidelines for apparently healthy and stable preterm infants up to a body weight of 1800 g. In other words, for moderate-to-late preterm infants (≥32 weeks GA) these guidelines may only account for the first 2–4 weeks of life. At higher body weights, current preterm formula probably provide too much energy for this target population.^15^ At the time of the study, the only alternative for the high calorie dense preterm formula were standard formulas. However, a standard formula did not show to be a good alternative and caused a higher increase in fat mass as compared to a low-calorie, but protein enriched post-discharge formula.^18^ Although the breastfed group showed a growth profile more in line with the reference values, the weight gain velocity during weeks 4–8 was still quite high. This might have been caused by the Nigerian policy to feed a breastfed infants at high frequency (at least every 3 h, at least 8 times a day, until satisfied), and over 1500 g of breastmilk per day.

Deficiencies of vitamin D and vitamin A were corrected in most formula-fed moderate-to-late preterm infants. In breastmilk-fed infants, vitamin D status improved mainly due to an increase in serum 25OHD_2_ which may have been derived from the vitamin D_2_ in the supplement Abidec (200–400 IU vitamin D_2_/day).^21^ In formula-fed infants, the increase was mainly in 25OHD_3_, as provided by the formula. Although Abidec also contains vitamin A (2000–4000 IU/day), this was not reflected in serum retinol of breastfed infants.

Despite a considerable iron intake (from supplement), formula-fed preterm infants showed a decrease in ferritin concentration (to ~100 µg/L) and Hb (to ~10 g/dL) during the first 10 weeks of life. These decreases were also seen in the breastmilk-fed infants, of whom surprisingly fewer (42% vs 82% in the formula group) received iron supplements at the age of 10 weeks (Supplementary Table s2). A postnatal (10–12 weeks) decline in Hb values to about 10 g/dL appears to be normal for most newborn infants and requires no therapy (physiological anemia of infancy). A faster decline (i.e., nadir at 4–6 weeks of age) to approximately 8 g/dL (anemia of prematurity) is associated with abnormal clinical signs and needs treatment.^31^ In our study, none of the formula fed infants reached this 8 g/dL, whereas 4 breastmilk-fed infants showed to have anemia of prematurity at the age of 10 weeks. The transient drop in Hb is caused by increased hepcidin (limiting iron absorption and releases from storage) transcription due to a higher ferritin and Hb status at birth.^41^ Besides, newborns have to switch from fetal (greater affinity to oxygen) to adult Hb (from birth onwards).^42^ Hb status will improve at a later age (from 3 to 6 months onwards), as shown in late preterm infants^41,42^ and Dutch post-discharge very preterm infants.^43^

As expected, concentrations of vitamins A and D and iron were low in the breast milk samples. Based on the vitamin A concentration in the Nigerian breastmilk (0.24 ± 0.08 µmol/L), all mothers were vitamin A deficient (cut-off value of 1.0 µmol/L), explaining the low vitamin A status of their newborns.^44^ The concentration of DHA suggests that the mothers’ diets contained a close to adequate amount of fish (or other DHA sources).

The strength of the study is the description of growth outcomes in breastmilk- and preterm formula-fed Nigerian moderate-to-late preterm infants under real life Nigerian conditions (including supplements, limited restrictions to milk intake, formula only on medical indication, and being at home). At the same time, all these practical conditions are limitations of this study. Nevertheless, the results are in line with expectations from literature and the study provides a clear view on current practice in Nigeria. The higher than recommended intake of formula, contributing to excessive intake of calories, is unfortunately general practice. The provision of vitamin and mineral supplements is both a limitation and a worry. The preterm formula provides all vitamins and minerals in sufficient amounts, and additional supplementation should only be based on biochemical parameters. Supplementation with vitamin D and K is generally recommended for breastmilk-fed infants. The finding that breastmilk-fed infants received less often iron supplements than formula-fed infants is surprising and unwanted.^43^ Finally, this study was not randomized which resulted in inclusion of more boys and multiple births in the formula-fed group compared to the breastmilk-fed counterparts. “The higher proportion of multiple births and SGA infants in the formula group introduced potential confounding, and the smaller sample size than anticipated reduced power”.

## Conclusion

Overall, this study shows that breastmilk feeding results in adequate growth of moderate-to-late preterm infants. When feeding these infants with a special preterm formula, weight gain is considerably faster than expected, whereas growth in length and HC are in line with the expectations. This outcome indicates that, following discharge, there is a need for adapted formulas to support normal growth of preterm infants who cannot be breastfed. Regular growth monitoring is necessary to monitor individual growth trajectory of preterm infants and adjust nutritional plan when necessary. The provision of vitamins A and D and iron in preterm formula appears to be sufficient. Vitamin A in breastmilk was too low for the present target population, probably because of maternal vitamin A deficiency. Vitamin D and iron are always low in breastmilk and should therefore be supplemented. Finally, overfeeding of formula-fed preterm infants, with supplements on top of that, appears to be common, even in a research setting, but should be prevented.

### Supplementary information

Supplementary Materials

## Data Availability

The data are not publicly available but can be provided upon reasonable request.
