# Supplementary material for: Growth and micronutrient status parameters of Nigerian preterm infants consuming preterm formula or breastmilk
Source: Pediatr Res. 2024 Jan 9;96(2):380–7. doi: 10.1038/s41390-023-02976-6 (PMC11343706; doi:10.1038/s41390-023-02976-6)
Supplement: Supplementary file 1 — Supplementary Materials [file 41390_2023_2976_MOESM1_ESM.pdf]

## Supplementary Materials

**Table s1**: Composition of Peak Baby Preterm per 100 kcal, as well as the recommended intakes per kg per day from the ESPGHAN (8).

| | **ESPGHAN 2010**[1] | **Peak Baby Preterm** |
|---|---|---|
| Protein hydrolysate (eq) | 3.5 – 4.0 | 3.2 |
| Fat (g) | 4.8 – 6.6 | 5.5 |
| α-linolenic acid (mg) | >55 | 78 |
| linoleic acid (mg) | 385 – 1540 | 560 |
| docosahexaenoic acid (mg) | 12-30 | 26.5 |
| arachidonic acid (mg) | 18-42 | 18.0 |
| Carbohydrates (g) | 11.6 – 13.2 | 9.5 |
| lactose (g) | | 7.8 |
| galacto-oligosaccharides (g) | | 0.1 |
| maltodextrin (g) | | 1.6 |
| Energy (kcal) | 110-135/kg BW | 80 |
| Na (mg) | 69 - 115 | 63 |
| K (mg) | 66 - 132 | 120 |
| Cl (mg) | 105 - 177 | 100 |
| Ca (mg) | 120 -140 | 124 |
| Mg (mg) | 8 - 15 | 10 |
| P (mg) | 60 - 90 | 69 |
| Fe (mg) | 2 - 3 | 1.67 |
| Cu (μg) | 100 - 132 | 94 |
| Mn (μg) | ≤27.5 | 24 |
| Zn (mg) | 1.1 – 2.0 | 1.1 |
| I (μg) | 11 - 55 | 31.4 |
| Se (μg) | 5 - 10 | 4.5 |
| F (μg) | 1.5 - 60 | 5.9 |
| Cr (ng) | 30 - 1230 | 90 |
| Mb (μg) | 0.3 – 5.0 | 3.6 |
| Vitamin A (μg-RE) | 400 - 1000 | 360 |
| Vitamin D (μg) | 20 – 25 | 6.7 |
| Vitamin E (mg α-TE) | 2.2 – 11 | 5.1 |
| Vitamin K (μg) | 4.4 – 28 | 9 |
| Vitamin B1 (μg) | 140 – 300 | 150 |
| Vitamin B2 (μg) | 200 – 400 | 220 |
| Niacin (μg-NE) | 380 – 5500 | 3750 |
| Vitamin B6 (μg) | 45 – 300 | 150 |
| Vitamin B12 (μg) | 0.1 – 0.77 | 0.27 |
| Folic acid (μg) | 35 – 100 | 45 |
| Pantothenic acid (μg) | 330 – 2100 | 900 |
| Biotin (μg) | 1.7 – 16.5 | 4.1 |
| Vitamin C (mg) | 11 – 46 | 20 |
| Choline (mg) | 8 – 55 | 20 |
| Inositol (mg) | 4.4 – 55 | 30 |
| Taurine (mg) | | 8 |
| Carnitine (mg) | | 2.6 |
| Nucleotides (mg) | | 5 |
| Osmolarity (mOsmol/L) | | ≤300 |

[1] Agostoni et al. Enteral nutrient supply for preterm infants. A comment of the ESPGHAN Committee of Nutrition. Journal of Pediatric Gastroenterology and Nutrition 2010. BW: body weight

**Table s2: Number of infants using additional supplements besides the breast milk or special preterm formula.**

| | Formula fed group (n=17) | | | Breast milk group (n=24) | | |
|---|---|---|---|---|---|---|
| | Age 2 wk | Age 6 wk | Age 10 wk | Age 2 wk | Age 6 wk | Age 10 wk |
| Abidec[1] | 9 (53) | 15 (88) | 10 (59) | 19 (79) | 23 (96) | 23 (96) |
| Calcimax | 7 (41) | 6 (35) | 5 (29) | 18 (75) | 20 (83) | 21 (88) |
| Ferrofer[2] | 3 (17) | 9 (53) | 14 (82) | 1 (4) | 4 (17) | 10 (42) |
| Folic acid | 11 (65) | 15 (88) | 15 (88) | 19 (79) | 22 (92) | 22 (92) |

Data presented as number and (%) of infants using supplements.
[1] Including Afrab Vite Multivitamins. [2] Including Astyfer, Ranferron and Orofer.

Abidec Multivitamin drops for babies (Medana Pharma SA, Sireadz, Poland) providing (in 0.3 ml/day): 2000 IU vitamin A, 200 IU vitamin D2, 0.5 mg vitamin B1, 0.2 mg vitamin B2, 0.25 mg vitamin B6, 2.5 mg niacin, and 25 mg vitamin C. In some cases Afrab Vite (Afrab-Chem Ltd., Lagos, Nigeria) multivitamin drops were used (0.6 ml/day): 4000 IU vitamin A, 400 IU vitamin D2, 1 mg vitamin B1, 0.4 mg vitamin B2, 0.5 mg vitamin B6, 5 mg niacin, and 25 mg vitamin C.
Calcimax Syrup (Vitabiotics Ltd, Lagos, Nigeria) providing (in 2.5 ml/day): 75 mg calcium, 12.5 mg magnesium, 0.75 mg zinc, and 100 IU vitamin D3.
Ferofer Syrup (Kwality Pharmaceuticals Ltd, Amritsar, India) providing (in 5 ml/day: 2x2.5 ml): 50 mg iron. In some cases Astyfer (per 10 ml: 47 mg iron, 5 mg vitamin B1, 3 mg vitamin B2, 2.5 µg vitamin B12, 0.5 mg folic acid, 25 mg niacin) or Ranferron-12 (per 5 ml: 41 mg iron, 5 µg vitamin B12, 0.5 mg folic acid) were used. Folic acid supplement, 2.5 mg daily.

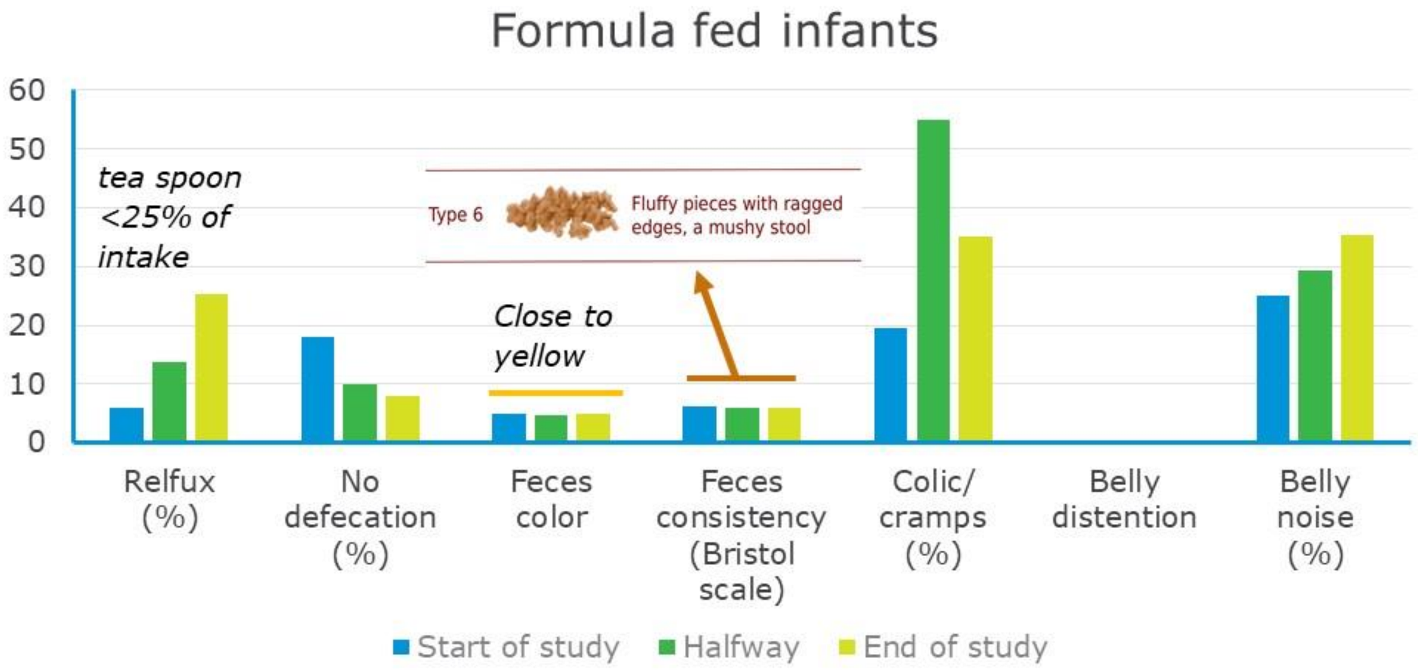
Formula fed infants
60
50
40
30
20
10
0
tea spoon
<25% of
intake
Type 6
Fluffy pieces with ragged
edges, a mushy stool
Close to
yellow
Relfux
(%)
No
defecation
(%)
Feces
color
Feces
consistency
(Bristol
scale)
Colic/
cramps
(%)
Belly
distention
Belly
noise
(%)
Start of study
Halfway
End of study

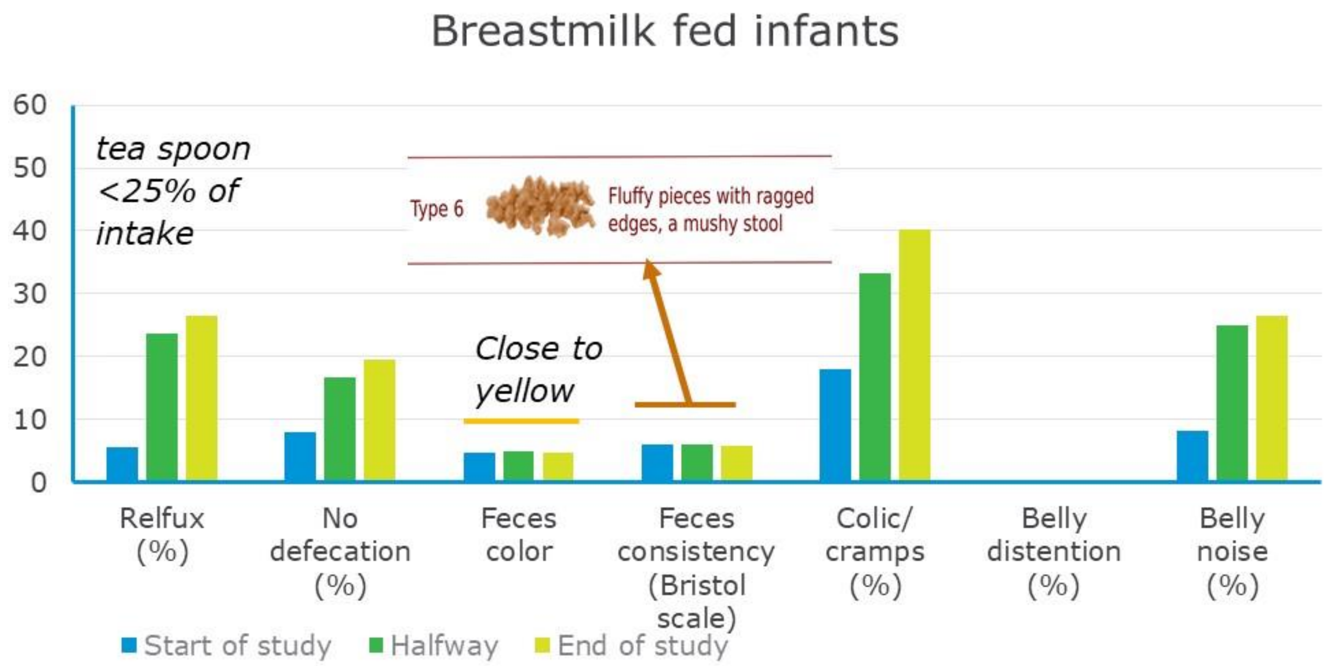


**Figure s1**: Tolerance indicators in exclusive breastmilk or preterm formula-fed Nigerian moderate-late preterm born infants at the age of 2 weeks (start of the study), 4 weeks (halfway) and 10 weeks of age (end).
